# Immunosenescence of the CD8^+^ T cell compartment is associated with HIV-infection, but only weakly reflects age-related processes of adipose tissue, metabolism, and muscle in antiretroviral therapy-treated HIV-infected patients and controls

**DOI:** 10.1186/s12865-015-0136-6

**Published:** 2015-11-26

**Authors:** Juliette Tavenier, Anne Langkilde, Thomas Huneck Haupt, Jens Henrik Henriksen, Frank Krieger Jensen, Janne Petersen, Ove Andersen

**Affiliations:** Optimed, Clinical Research Centre, Copenhagen University Hospital, Hvidovre, Kettegård Alle 30, DK-2650 Hvidovre, Denmark; Department of Clinical Physiology and Nuclear Medicine, Copenhagen University Hospital, Hvidovre, Kettegård Alle 30, DK-2650 Hvidovre, Denmark; Department of Radiology, Copenhagen University Hospital, Hvidovre, Kettegård Alle 30, DK-2650 Hvidovre, Denmark; Section of Biostatistics, Department of Public Health, Faculty of Health and Medical Sciences, University of Copenhagen, Øster Farimagsgade 5, 1014 Copenhagen K., Denmark; Department of Infectious Diseases, Copenhagen University Hospital, Hvidovre, Kettegård Alle 30, DK-2650 Hvidovre, Denmark

**Keywords:** Immunosenescence, Ageing, HIV, PD-1, KLRG1

## Abstract

**Background:**

Despite effective antiretroviral therapy (ART), HIV-infected patients exhibit systemic inflammation, early onset of age-related diseases, and features of immunosenescence. The role of inflammation in the development of age-related diseases is widely recognized. However, the role of immunosenescence is not well established. Studying immunosenescence in HIV-infection could give insight into its role in ageing processes. In this cross-sectional study, we aimed to investigate whether ART-treated HIV-infected patients exhibit immunosenescence; and whether immunosenescence is associated with age-related processes of inflammation, metabolism, adipose tissue, and muscle. T cell immunosenescence and exhaustion were assessed by flow cytometry analysis of CD8^**+**^ cells from 43 ART-treated HIV-infected patients (HIV^+^) and ten Controls using markers of differentiation: CD27/CD28; maturation: CD27/CD45RA; senescence: killer cell lectin-like receptor G1 (KLRG1); and exhaustion: programmed death-1 (PD-1). Relationships between CD8^**+**^ T cell immunosenescence, exhaustion, and age-related processes were assessed using linear regressions.

**Results:**

HIV-infection was strongly associated with more highly differentiated and mature CD8^**+**^ T cell phenotypes. PD-1 and KLRG1 expression did not differ between HIV^+^ and Controls, but depended on differentiation and maturation stages of the cells. CD8^**+**^ T cell maturation was associated with age. KLRG1 expression was associated with age, metabolic syndrome, visceral adipose tissue, and high muscle mass. PD-1 expression was not associated with age-related parameters.

**Conclusions:**

HIV-infection strongly affected CD8^**+**^ T cell differentiation and maturation, whereas age-related processes were only weakly associated with immune parameters. Our findings suggest that, in contrast to inflammation, immunosenescence appears to be highly dependent on HIV-infection and is only to a small extent associated with age-related parameters in well-treated HIV-infection.

**Electronic supplementary material:**

The online version of this article (doi:10.1186/s12865-015-0136-6) contains supplementary material, which is available to authorized users.

## Background

The world’s population is ageing rapidly. The population of individuals aged 60 or over is the fastest growing, and it is estimated to represent over 20 % of the world’s population by 2050 [1]. As a result, the prevalence of age-related diseases–such as cardiovascular diseases, metabolic syndrome and diabetes, loss of muscle mass, and cancer–is increasing. It is now evident that ageing is associated with a state of chronic low-grade inflammation known as “inflammaging”, and that most age-related diseases are–in part–caused by inflammaging. Inflammaging is characterized by elevated levels of pro-inflammatory biomarkers such as interleukin-6 (IL-6), tumour necrosis factor alpha, and C-reactive protein. However, it is not well known what causes the increased inflammation [2, 3].

As immune cells are major producers of inflammatory proteins, inflammaging could originate from the changes that occur to immune cells during ageing, termed immunosenescence [4]. Immunosenescence is characterized by decreased output of naïve T cells following thymic involution, and accumulation of highly differentiated memory CD8^**+**^ T cells as a result of repeated antigenic stimulation and chronic viral infections such as cytomegalovirus (CMV)-infection [5]. This results in an inversion of the CD4:CD8 T cell ratio [5, 6]. The highly differentiated T cells are senescent in that they lose proliferative capacity, rendering them inefficient against pathogens. However, they are able to produce elevated amounts of pro-inflammatory cytokines [7–9].

Antiretroviral therapy (ART)-treated HIV-infected patients are characterised by earlier onset of age-associated diseases like cardiovascular diseases; features of immunosenescence; inflammaging; and loss of T cell effector functions, referred to as T cell exhaustion [10–14]. Moreover, ART is associated with development of lipodystrophy, a syndrome of adipose tissue redistribution characterized by loss of subcutaneous adipose tissue and gain of visceral adipose tissue (VAT), and which resembles the changes in body composition that occur with age [15, 16].

While immunosenescence is well-documented with age, and is increasingly reported in treated HIV-infection, it is not known whether these immunological changes are involved in age-associated disease processes. Studying the associations between immunosenescence and clinical age-related processes in HIV-infected patients could therefore yield insight into the role of immunosenescence in disease development. In this study we therefore wanted to investigate whether ART-treated HIV-infected patients exhibit immunosenescence; and whether this immunosenescence is associated with age-related processes of inflammation, metabolism, adipose tissue, and muscle.

## Methods

### Study design and participants

Between November 2010 and October 2012, 75 participants were included in this cross-sectional study: 60 patients with HIV-infection (HIV^+^) and 15 healthy men of similar age (Controls). HIV^+^ were recruited from the Department of Infectious Diseases, and Controls were recruited by advertisement at Copenhagen University Hospital, Hvidovre, Denmark. Inclusion criteria were: male sex; white ethnicity; > 18 years old; testing negative for hepatitis B and C; no intravenous drug use; and no current immunomodulating, lipid-lowering, anti-diabetic, or endocrinologic treatment. HIV^+^ were also required to have plasma HIV-RNA <400 copies/mL; ART for at least 12 months; and CD4^**+**^ T cell counts ≥200 cells/μL. The relationships between inflammatory biomarkers, adipose tissue distribution and low muscle mass in this cohort have been investigated [17]. Moreover, microdialysis results from 18 of the study participants have been published in a methodological study [18]. The study was approved by the local ethics committee of the Capital Region of Denmark (H-4-2010-045), the Danish Data protection agency (2010-41-4952), and was conducted in accordance with the Declaration of Helsinki. All participants gave written informed consent.

### Plasma inflammation markers and blood sample measurements

Plasma levels of the inflammation markers soluble urokinase plasminogen activator receptor (suPAR) and IL-6 were measured by enzyme-linked immunosorbent assays: suPARnostic (ViroGates A/S, Birkerød, Denmark) and Quantikine HS-IL-6 (R&D Systems, Minneapolis, MN). CD4^+^, CD8^+^ T cell counts, and HIV-RNA were routine measurements by the Department of Clinical Biochemistry, Copenhagen University Hospital, Hvidovre, Denmark. Peripheral blood mononuclear cells were isolated by centrifugation using BD Vacutainer cell preparation tubes (BD Biosciences, San Jose, CA) and cryopreserved.

### Flow cytometric analysis of CD8^+^ T cells

Cryopreserved peripheral blood mononuclear cells from all participants were thawed and washed in complete RPMI 1640 media supplemented with GlutaMAX (Life Technologies, Grand Island, NY), 10 % fetal bovine serum, 100 U/mL penicillin, and 100 μg/mL streptomycin. 1.5 million cells were resuspended in 100 μL PBS and stained with the Zombie NIR Fixable Viability Kit (BioLegend, San Diego, CA) for 30 min. Cells were washed and resuspended in 10 % heat inactivated human serum in PBS for 20 min for Fc receptor blocking. Cells were incubated for 30 min with titrated anti-CD3-FITC, anti-CD8-PerCp-Cy5.5, anti-CD27-APC, anti-CD28-PE-Cy7 or anti-CD45RA-PE-Cy7 (BD Biosciences), and anti-PD-1-PE or anti-KLRG1-PE (BioLegend) antibodies and washed in FACS buffer (2 mM EDTA (Life Technologies) and 0.5 % bovine serum albumin (Miltenyi Biotec, Cologne, Germany) in PBS). Matching isotype and unstained controls were included for each participant. Compensations were generated for each fluorochrome using CompBeads (Anti-Mouse Ig, κ) (BD Biosciences). For each sample, 50 000 to 100 000 CD8^+^ events were recorded on a FACSCanto II flow cytometer (BD Biosciences). HIV- and lipodystrophy-status of the participants were concealed until after data analysis. Data were analysed using Cytobank [19].

CD8^**+**^ T cells could be subtyped into four maturation subsets based on CD27 and CD45RA expression: naïve (T_N_: CD27^+^CD45RA^+^); central memory (T_CM_: CD27^+^CD45RA^−^); effector memory (T_EM_: CD27^−^CD45RA^−^); or effector memory re-expressing CD45RA (T_EMRA_: CD27^−^CD45RA^+^) [20, 21]. By evaluating the expression of CD27 and CD28, CD8^**+**^ T cells could also be subtyped into four differentiation subsets: early differentiated (T_ED_: CD27^+^CD28^+^); intermediate differentiated (T_ID_: CD27^+^CD28^−^); late differentiated (T_LD_: CD27^−^CD28^−^); or CD27^−^CD28^+^ [8]. The expression of the exhaustion marker PD-1 and of the senescence marker KLRG1 was evaluated in each of the maturation and differentiation subsets and in the total CD8^**+**^ T cell population [22].

### Assessment of body composition

Anthropometric measurements comprised body weight, height, and waist and hip circumferences, measured as in [15]. Abdominal VAT mass at the level of L4 was derived from computed tomography scans (Somatom Sensation 10, Siemens, Germany). Body composition was determined by dual energy X-ray absorptiometry (Norland XR-36, Gammatec A/S, Værløse, Denmark). Fat mass index (FMI) was calculated as: total fat mass/height^2^ (kg/m^2^). Leg lean mass index (*l*LMI) was calculated as: leg lean mass/height^2^ (kg/m^2^). Lean mass was used as a surrogate marker for muscle mass as lean mass is principally made of skeletal muscle. This is especially true for lean mass in the extremities [23].

### Assessment of metabolism

Fasting glucose levels were routine measurements by the Department of Clinical Biochemistry. Fasting plasma levels of insulin were measured using Immulite 2000 System (Siemens, NY, USA) and insulin resistance was calculated using the homeostasis model assessment (HOMA-IR) [24]. Metabolic syndrome was defined according to the 2009 consensus definition for high risk populations [25]. The assessment of metabolic syndrome was based on the presence of at least three of the five following criteria: elevated waist circumference (≥94 cm, as recommended for high risk populations); elevated triglycerides (≥1.7 mmol/L); reduced HDL-C (<1.0 mmol/L); elevated blood pressure (systolic ≥130 or diastolic ≥85 mm Hg, or anti-hypertensive treatment); elevated fasting glucose (≥100 mg/dL).

### Assessment of lipodystrophy

Lipodystrophy status and the type of lipodystrophy were evaluated by clinical evaluation of the adipose tissue distribution of several subcutaneous regions (i.e. face, retroauricular and dorsocervical regions, upper arms, thighs, buttocks, and abdomen) and of the visceral region, as in [15]. HIV^+^ were characterized as lipodystrophic or non-lipodystrophic. Patients with lipodystrophy were further characterized as lipohypertrophic, lipoatrophic, or having mixed type lipodystrophy.

### Statistical analysis

Comparisons between HIV^+^ and Controls were performed using unpaired Student’s *t*-test or Wilcoxon two-sample test where appropriate. For categorical parameters, Chi-squared test or Fisher’s exact test were used. To investigate the effect of HIV-infection on CD8^**+**^ T cell phenotypes, we used unpaired Student’s *t*-test or Wilcoxon two-sample test and linear regression analyses. The associations between HIV-and ART-related variables and CD8^**+**^ T cell phenotypes were investigated in the HIV^+^ group using multiple linear regression analyses adjusted for age. The associations between age, inflammation, metabolism, body composition, and CD8^**+**^ T cell phenotypes were investigated in the HIV^+^ group using simple and multiple linear regression analyses adjusted for age, HIV-, ART-duration, and current ART. The T_CM_, T_EM_, and T_EMRA_ subsets were grouped into one Memory group. Thus CD8^**+**^ T cell maturation was defined as either an antigen-inexperienced naïve (T_N_) or an antigen-experienced Memory phenotype. CD8^**+**^ T cell differentiation was defined as either an undifferentiated CD28^+^ phenotype composed of T_ED_ and CD27^−^CD28^+^ cells, or a highly differentiated CD28^−^ phenotype composed of T_ID_ and T_LD_ cells. The loss of CD28 expression is considered to identify a group of cells characterized by functional changes and replicative senescence [9]. By grouping the individual subsets according to relevant biological and functional characteristics, we could reduce the number of analyses and simplify the interpretation. Nonetheless, data on the individual subsets are available as Additional files. Due to the compositional nature of the data, only the estimates of the Memory and CD28^−^ cells are presented in analyses for group sizes. The estimates for T_N_ cells are the opposite of the estimates for Memory cells since their proportions add up to 100 %, and the same is true for CD28^+^ and CD28^−^ cells. If the residuals were not normally distributed, the parameter was log-transformed to fulfil model requirements using log_2_(x). Beta estimates (β) for log-transformed parameters were back transformed using (2^β^ -1) × 100, and thereby shown as percent change in outcome per unit increase of the covariate.

Statistical significance was defined as a *P*-value < 0.05. Analyses were performed using Statistical Analysis Systems (SAS) version 9.4 (SAS Institute, Cary, NC, USA). Graphs were made with GraphPad Prism version 6 (GraphPad Software, San Diego, CA, USA).

## Results

**Fig. 1:**
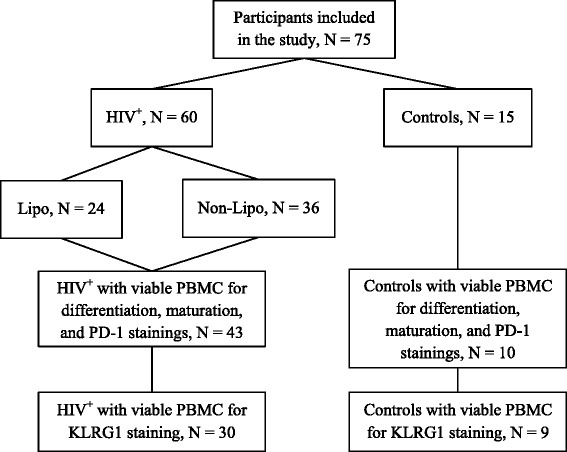
Flowchart of the study participants. Abbreviations: HIV^+^: HIV-infected patients, KLRG1: killer cell lectin-like receptor G1, Lipo: lipodystrophic, Non-Lipo: non-lipodystrophic, PBMC: peripheral blood mononuclear cell, PD-1: programmed death-1

Viable PBMCs were available from 53 of the 75 participants, and only these participants were included in the study (Fig. 1). Flow cytometry analysis of maturation and differentiation markers, and PD-1, was done for all 53 participants, whereas KLRG1 analyses was done for only 39 participants since not enough cells were available for all participants.

General characteristics of the study participants are summarized in Table 1. HIV^+^ had significantly higher CD8^**+**^ T cell numbers (*P* < 0.0001) and lower CD4:CD8 ratio (*P* < 0.0001) than Controls. Smoking prevalence (*P* = 0.05), HOMA-IR index (*P* = 0.05), and suPAR levels (*P* = 0.02) were significantly higher in HIV^+^ compared to Controls. Age and body composition did not differ between HIV^+^ and Controls.

**Table 1:** General characteristics of the study participants

	Controls		HIV^+^	
	*N* = 10		*N* = 43	
	Median or n	IQR or %	Median or n	IQR or %
Demography and lifestyle				
Age (years)	48.1	41.9–62.3	49.8	43.3–55.2
Smoking	0	0 %	14*	32.6 %
Body composition				
Lipodystrophy	—	—	14	32.6 %
BMI (kg/m^2^)	25.4	24.7–28.6	25.1	23.0–27.7
FMI (kg/m^2^)	5.2	4.6–7.5	4.6	3.4–6.4
VAT (cm^2^)	111.6	93.5–157.5	150.8	115.0–212.2
*l*LMI (kg/m^2^)	6.4	6.2–6.8	6.1	5.7–6.6
Metabolic parameters				
HOMA-IR	0.5	0.5–1.3	1.2*	0.6–2.4
Metabolic syndrome	1	10 %	20	46.5 %
Biomarkers of inflammation				
IL-6 (pg/mL)	1.1	0.7–2.4	1.6	1.1–2.6
suPAR (ng/mL)	1.8	1.5–2.1	2.2*	1.8–2.8
HIV- and immune-related parameters				
HIV duration (years)	—	—	14	6.6–21.0
ART duration (years)	—	—	10.2	4.4–14.8
Current PI treatment	—	—	13	30.2 %
Current NRTI treatment	—	—	43	100 %
Current NNRTI treatment	—	—	28	65.1 %
Other current ART	—	—	4	9.30 %
HIV RNA ≤20 copies/mL	—	—	39	90.7 %
CD4^**+**^ T cell count (cells/μL)	729	674–880	580	403–820
CD8^**+**^ T cell count (cells/μL)	373	281–440	828***	618–1120
CD4:CD8 ratio	2.0	1.8–3.0	0.7***	0.5–1.0

Continuous measures are listed as: median and interquartile range. Categorical variables are listed as: number of participants and percentage

Abbreviations: *BMI* body mass index, *ART* antiretroviral therapy, *FMI* fat mass index, *HOMA-IR* homeostatic model assessment of insulin resistance, *IL-6* interleukin-6, *IQR* interquartile range, *lLMI* leg lean mass index, *NRTI* nucleoside reverse-transcriptase inhibitors, *NNRTI* non-nucleoside reverse-transcriptase inhibitor, *PI* protease inhibitor, *suPAR* soluble urokinase plasminogen activator receptor, *VAT* visceral adipose tissue

The bold values are statistically significant at * *P* < 0.05, ** *P* < 0.01, *** *P* < 0.001

### CD8^+^ T cell compartment re-modulation in HIV-infection

**Fig. 2:**
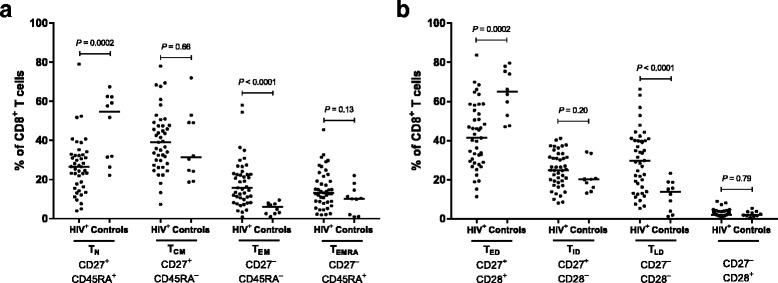
CD8^+^ T cell maturation and differentiation in HIV^+^ and in Controls. **a** Proportions of CD8^+^ T cells in each maturation subset: naïve (T_N_), central memory (T_CM_), effector memory (T_EM_), or effector memory re-expressing CD45RA (T_EMRA_) **b** Proportions of CD8^+^ T cells in each differentiation subset: early differentiated (T_ED_), intermediate differentiated (T_ID_), late differentiated (T_LD_), or CD27^−^CD28^+^. HIV^+^ (*N* = 43) and Controls (*N* = 10). Medians are shown as *horizontal bars. P*-values were determined using unpaired Student’s *t*-test or Wilcoxon two-sample test

Lipodystrophy was not associated with changes in CD8^**+**^ T cell phenotype (Additional file 1 and Additional file 2). Therefore, patients with and without lipodystrophy were grouped (HIV^+^) in further analyses. CD8^**+**^ T cell maturation phenotypes: naïve (T_N_), central memory (T_CM_), effector memory (T_EM_), or effector memory re-expressing CD45RA (T_EMRA_), were determined based on CD27 and CD45RA expression. HIV^+^ had significantly lower proportions of T_N_ cells (*P* < 0.001) and significantly higher proportions of T_EM_ cells (*P* = 0.002) than Controls (Fig. 2a). CD8^**+**^ T cell differentiation phenotypes: early (T_ED_), intermediate (T_ID_), late (T_LD_) differentiated, or CD27^−^CD28^+^, were determined based on CD27 and CD28 expression. HIV^+^ had significantly lower proportions of T_ED_ cells (*P* < 0.001) and significantly higher proportions of T_LD_ cells (*P* < 0.001) than Controls (Fig. 2b). We investigated whether these changes were mediated by differences in age or age-related processes using multiple regression analyses. The differences in CD8^+^ T cell subset distribution between HIV^+^ and Controls were primarily related to HIV or ART. Adjusting for age, inflammation, metabolism, or body composition, hardly affected the associations between HIV-infection and CD8^+^ T cell maturation and differentiation (Additional file 3).

Moreover, we investigated whether HIV-, ART-duration or current ART was associated with differences in CD8^+^ T cell subset distribution in HIV-infected patients using linear regressions adjusted for age. Current use of protease inhibitors was significantly associated with higher proportions of T_EM_ (*P* = 0.002) and T_LD_ (*P* = 0.002), and lower proportions of T_N_ (*P* = 0.007) and T_ED_ (*P* = 0.04) CD8^+^ T cells. Current use of non-nucleoside reverse-transcriptase inhibitors was associated with higher proportions of T_ID_ (*P* = 0.01). HIV- (*P* = 0.36–0.99) or ART-duration (*P* = 0.07–0.94) did not have an effect on CD8^+^ T cell subset sizes.

### PD-1 and KLRG1 expression in HIV-infection

**Fig. 3:**
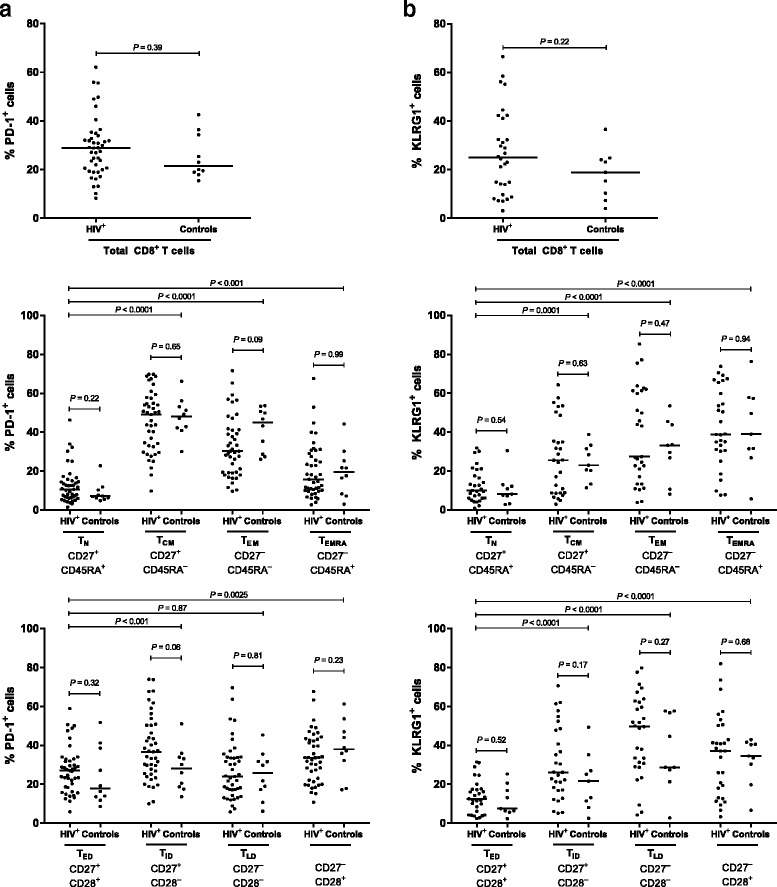
CD8^+^ T cell exhaustion and senescence in HIV^+^ and in Controls. **a** Proportions of PD-1^+^ cells in total CD8^+^ T cells and in CD8^+^ T cell subsets in HIV-infection and in Controls. HIV^+^ (*N* = 43) and Controls (*N* = 10). **b** Proportions of KLRG1^+^ cells in total CD8^+^ T cells and in CD8^+^ T cell subsets in HIV^+^ and in Controls. HIV^+^ (*N* = 30) and Controls (*N* = 9). Medians are shown as *horizontal bars. P*-values were determined using unpaired Student’s *t*-test or Wilcoxon two-sample test

CD8^**+**^ T cell senescence and exhaustion were assessed on the basis of KLRG1 and PD-1 expression, respectively. HIV-infection did not have a significant effect on PD-1 and KLRG1 expression, neither in the total CD8^**+**^ T cell population or in the subsets (*P* = 0.06–0.94) (Fig. 3). Instead, PD-1 and KLRG1 expression depended on the maturation and differentiation stages of the cells. PD-1 expression was highest in intermediate subsets (Fig. 3a). KLRG1 expression was highest in highly differentiated subsets (Fig. 3b). Moreover, there was a significant association between proportions of PD-1^+^ and KLRG1^+^ CD8^**+**^ T cells across both groups, the proportion of KLRG1^+^CD8^**+**^ T cells was multiplied by 2.7 % for each 1 % increase in the proportion of PD-1^+^CD8^**+**^ T cells (Estimate = 2.72 %; *P* = 0.02, 95 % CI = 0.45–5.04). We did not find any association between HIV-, ART- duration, or ART and changes in KLRG1 or PD-1 expression (*P* = 0.06–0.89).

### Associations between CD8^+^ T cell subsets, age, and age-related parameters

**Fig. 4:**
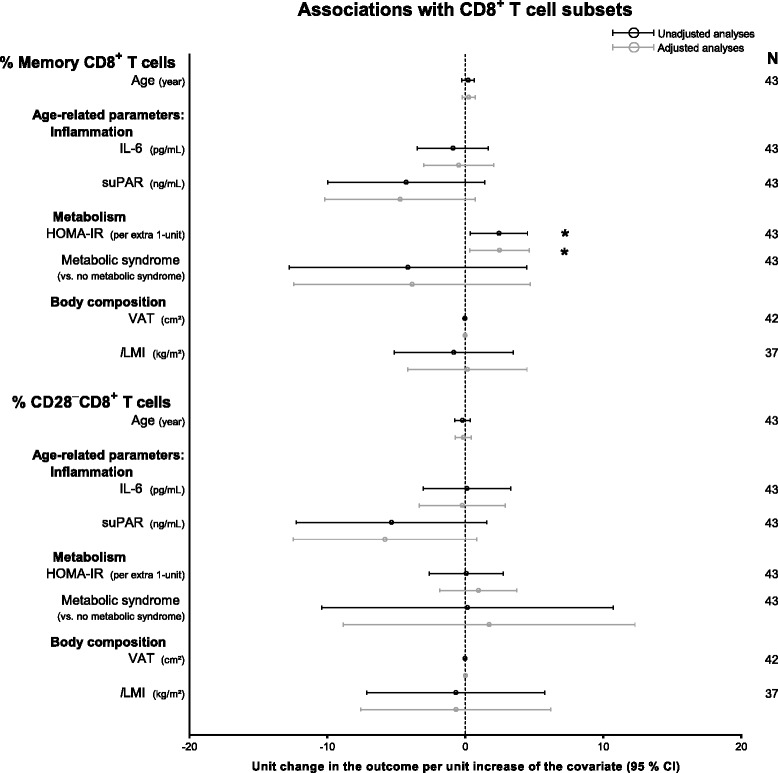
Unadjusted and adjusted associations between Memory and CD28^−^CD8^+^ T cells and ageing parameters in HIV^+^. Age was adjusted for HIV-, ART-duration, and current ART; IL-6, suPAR, HOMA-IR, metabolic syndrome, VAT, and *l*LMI were adjusted for age, HIV-, ART-duration, and current ART. Estimates are shown as unit increase in the outcome per unit increase of the covariate. The dashed line marks an estimate change of 0 units. *P < 0.05. Abbreviations: HOMA-IR: homeostatic model assessment of insulin resistance; IL-6: interleukin-6; *l*LMI: leg lean mass index; suPAR: soluble urokinase plasminogen activator receptor; VAT: visceral adipose tissue

The aim of the study was to investigate changes within the CD8^+^ T cell compartment in well-treated HIV-infected patients, and further to investigate whether these changes were associated with age-related parameters. In contrast to the virus, which is thought to affect subsets with specific effector functions, we expected the ageing process to have a more general effect on the CD8^+^ T cell population. Therefore, we grouped T_CM_, T_EM_, and T_EMRA_ subsets into one Memory group; T_ED_ and CD27^−^CD28^+^ cells into one CD28^+^ group; and T_ID_ and T_LD_ into one CD28^−^ group. When grouping the subsets, HIV-infection remained associated with lower proportions of T_N_ and CD28^+^, and higher proportions of Memory (*P* = 0.0002) and CD28^−^ (*P* = 0.0003) CD8^+^ T cells. We investigated the associations between CD8^+^ T cell distribution, age, and age-related parameters in the HIV^+^ group in simple and adjusted analyses (Fig. 4). Analyses for the individual subsets are available in Additional file 4. HOMA-IR index was significantly associated with higher proportions of Memory (Unadjusted *P* = 0.02, adjusted *P* = 0.03), mainly T_EM_ cells (Unadjusted *P* = 0.02, adjusted *P* = 0.003), and lower proportions of T_N_ cells (Unadjusted *P* = 0.02, adjusted *P* = 0.03) (Fig. 4 and Additional file 4). We found no significant associations between memory or CD28^−^ CD8^+^ T cell group sizes with age, inflammation or VAT overall (Fig. 4). However, at the individual subset level, IL-6 levels were associated with changes in the proportions of T_ID_, and VAT was associated with lower proportions of T_EMRA_ CD8^+^ T cells (Additional file 4).

### Associations between PD-1 and KLRG1 expression, age and age-related parameters

**Fig. 5:**
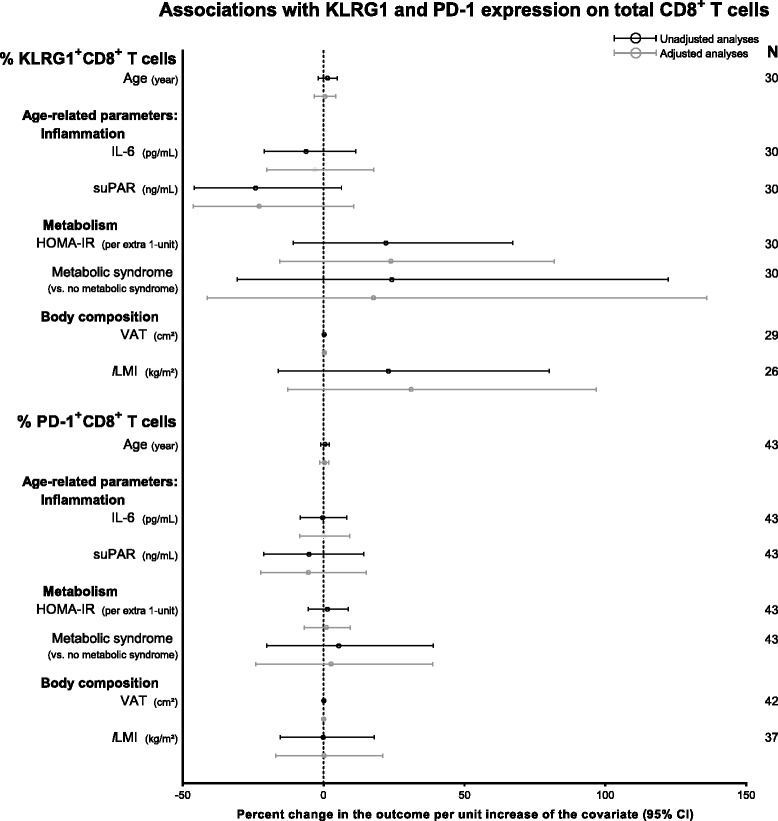
Unadjusted and adjusted associations between KLRG1^+^ and PD-1^+^CD8^+^ T cells and ageing parameters in HIV^+^. Age was adjusted for HIV-, ART-duration, and current ART; IL-6, suPAR, HOMA-IR, metabolic syndrome, VAT, and *l*LMI were adjusted for age, HIV-, ART-duration, and current ART. Parameters were transformed using log_2_(x). Estimates and 95 % CI are back transformed using (2^β^-1) × 100 and shown as percent change in the outcome per unit increase of the covariate. The *dashed line* marks an estimate change of 0 %. Abbreviations: HOMA-IR: homeostatic model assessment of insulin resistance; IL-6: interleukin-6; KLRG1: killer cell lectin-like receptor G1; *l*LMI: leg lean mass index; PD-1: programmed death-1; suPAR: soluble urokinase plasminogen activator receptor; VAT: visceral adipose tissue

We found PD-1 and KLRG1 expression to vary according to subset differentiation and maturation, but not to HIV-infection (Fig. 3). Thus, we wanted to investigate whether PD-1 and KLRG1 expression in total CD8^**+**^ T cells were associated with age and age-related processes in the HIV^+^ group in simple and adjusted regression analyses. When grouping the subsets, HIV-infection was not associated with changes in KLRG1 or PD-1 expression (*P* = 0.14–0.87). There were no associations between KLRG1 or PD-1 expression in total CD8^**+**^ T cells and age-related parameters (Fig. 5).

Within each CD8^**+**^ T cell subset, there were still large variations in the number of KLRG1^+^ and PD-1^+^ cells. We therefore also investigated whether age and age-related processes were associated with PD-1 and KLRG1 expression in specific CD8^**+**^ T cell groups: T_N_ and Memory, and CD28^+^ and CD28^−^ cells. In participants with metabolic syndrome, the proportion of KLRG1^+^CD28^−^ cells increased by 14 % in unadjusted analyses, and by 18 % in adjusted analyses (Unadjusted *P* = 0.05, adjusted *P* = 0.04), compared to those without metabolic syndrome (Table 2). *l*LMI was associated with an increase in the proportions of KLRG1^+^T_N_ cells (*P* = 0.02). VAT was associated with an increase in the expression of KLRG1 in CD28^+^ (Unadjusted *P* = 0.02, adjusted *P* = 0.03) and CD28^−^ cells (Adjusted *P* = 0.04). Associations of KLRG1 expression and metabolic syndrome, VAT and *l*LMI were also observed in the individual subsets (Additional file 5).

**Table 2:** Unadjusted and adjusted associations between KLRG1^+^CD8^+^ T cell groups and ageing parameters in HIV^+^

	Maturation - CD27/CD45RA				Differentiation - CD27/CD28			
	% KLRG1^+^ T_N_^a^		% KLRG1^+^ Memory^a^		% KLRG1^+^CD28^+a^		% KLRG1^+^CD28^−^	
	Estimate	95 % CI	Estimate	95 % CI	Estimate	95 % CI	Estimate	95 % CI
Age (per year extra)	−0.84	−4.32 – 2.77	1.12	−2.19 – 4.53	1.54	−1.73 – 4.93	0.05	−0.81 – 0.92
	−0.16	−4.22 – 4.07	0.32	−3.53 – 4.32	0.49	−3.16 – 4.28	−0.24	−1.24 – 0.76
IL-6 (per extra pg/mL)	−0.24	−17.69 – 20.92	−8.07	−22.94 – 9.68	3.27	−13.80 – 23.71	−0.84	−5.53 – 3.85
	−2.98	−21.71 – 20.23	−7.08	−23.90 – 13.47	9.10	−9.84 – 32.03	0.11	−5.16 – 5.38
suPAR (per extra ng/mL)	−13.66	−40.90 – 26.14	−21.72	−44.54 – 10.50	−18.83	−42.12 – 13.82	−5.62	−14.38 – 3.14
	−15.63	−43.65 – 26.30	−20.25	−45.09 – 15.84	−17.17	−41.55 – 17.38	−4.90	−14.36 – 4.56
HOMA-IR (per extra 1-unit)	16.39	−17.36 – 63.93	18.91	−13.42 – 63.30	27.27	−6.34 – 72.93	6.02	−1.96 – 14.01
	35.74	−9.23 – 103.01	22.91	−16.71 – 81.38	27.45	−10.90 – 82.29	6.98	−2.65 – 16.16
Metabolic syndrome (vs. no metabolic syndrome)	23.25	−34.76 – 132.83	46.43	−17.97 – 161.42	70.88	−1.41 – 196.17	14.31*	0.10 – 28.53
	48.94	−29.02 – 212.55	41.31	−29.88 – 184.78	61.77	−15.73 – 210.55	17.59*	0.73 – 34.45
VAT (per extra cm^2^)	−0.10	−0.55 – 0.35	0.28	−0.12 – 0.70	0.45*	0.06 – 0.84	0.10	−0.00 – 0.20
	0.14	−0.54 – 0.82	0.28	−0.34 – 0.90	0.62*	0.07 – 1.16	0.16*	0.01 – 0.31
*l*LMI (per extra kg/m^2^)	66.47*	10.22 – 151.43	36.88	−5.52 – 98.30	22.54	−20.06 – 87.86	7.92	−2.88 – 18.71
	59.19	−0.55 – 154.80	45.52	−1.35 – 114.65	33.83	−13.85 – 107.92	10.09	−1.22 – 21.39

Adjusted analyses are in grey. Age was adjusted for HIV-duration, ART-duration, and current ART; IL-6, suPAR, HOMA-IR, metabolic syndrome, VAT, and *l*LMI were adjusted for age, HIV-duration, ART-duration, and current ART

Abbreviations: *HOMA-IR* homeostatic model assessment of insulin resistance, *IL-6* interleukin-6, *KLRG1* killer cell lectin-like receptor G1, *lLMI* leg lean mass index, *suPAR* soluble urokinase plasminogen activator receptor, *T*_*N*_ naïve T cell, *VAT* visceral adipose tissue

The bold values are statistically significant at * *P* < 0.05

^a^Parameters were transformed using log_2_(x). Estimates and confidence intervals are back transformed using (2^β^-1) × 100, and shown as percent change in the outcome per unit increase of the covariate

We found no associations between PD-1 expression and age-related parameters (Additional file 6 and Additional file 7).

## Discussion

We investigated whether CD8^**+**^ T cell maturation, differentiation, senescence, and exhaustion differed between well-treated HIV-infected patients and Controls; and whether these immunological changes were associated with age and age-related processes of inflammation, metabolism, adipose tissue, or muscle. HIV^+^ had significantly higher levels of CD8^**+**^ T cells with mature and highly differentiated phenotypes, but not higher levels of CD8^**+**^ T cells expressing KLRG1 or PD-1 compared to Controls. Instead, KLRG1 and PD-1 expression were highly dependent on CD8^**+**^ T cell maturation and differentiation. In contrast, age-related processes were only weakly and inconsistently associated with CD8^**+**^ T cell phenotypes.

In agreement with previous studies, HIV-infection was strongly associated with lower proportions of relatively undifferentiated and naïve CD8^**+**^ T cells, higher proportions of highly differentiated and mature CD8^**+**^ T cells, and an inverted CD4:CD8 T cell ratio (<1) [12, 26]. These features are characteristic of the immunosenescence phenotype previously described in healthy elderly individuals [4]. However, while HIV-infection is characterized by increased proportions of intermediate T_EM_ CD8^**+**^ T cells, ageing is characterized by increased proportions of more highly differentiated T_EMRA_ CD8^**+**^ T cells, often attributed to CMV infection [21, 26–28]. This indicates that there are subtle differences in the way CD8^**+**^ T cell subsets are affected by HIV-infection and age.

HIV^+^ patients did not normalize their CD8^**+**^ maturation and differentiation pattern despite successful ART, indicating that HIV viral load is not the only driver of CD8^**+**^ T cell phenotypic abnormalities. Other explanations include disturbed thymic function [29, 30], ongoing replication in HIV-reservoirs [31], co-infections by other chronic viruses, and bacterial translocation in the gut [32]. The effect of HIV-infection on CD8^**+**^ T cell maturation and differentiation did not appear to be mediated by age-related processes, since the estimates for the associations between HIV-infection and T cell phenotypes were unchanged when adjusted.

Previous studies have investigated the association between lymphocytes phenotypes and age-associated clinical outcomes, but with varying results. Highly differentiated CD28^−^CD8^**+**^ T cells were associated with frailty in older women [33]. Others have found associations of CD8^**+**^ T cell differentiation and maturation with obesity in children [34]. CD8^+^ T cell maturation was associated with HOMA-IR index. However, we found HIV-infection to be associated with both higher proportions of memory CD8^+^ T cells, and with elevated HOMA-IR index. Thus HIV-infection could be a confounder of this association. We did not observe robust associations between CD8^**+**^ T cell maturation and differentiation and body composition. Our results are in agreement with Erlandson et al*.* who reported an association of low physical function with inflammation, but not with highly differentiated CD28^−^ T cells, in HIV-infected patients, [35]. Moreover, Wallet et al. reported neither elevated inflammation nor higher proportions of senescent CD57^+^ CD4^+^ and CD8^+^ T cells to be associated with physical function in older HIV-infected patients [36].

HIV-infection was not associated with higher PD-1 or KLRG1 expression in CD8^**+**^ T cells. However, PD-1 and KLRG1 expression depended on differentiation and maturation stages of the cells. Consistent with previous studies, PD-1 expression was highest in intermediately differentiated and mature subsets, and KLRG1 expression was highest in highly differentiated and mature subsets [37–39]. PD-1 expression has been reported to be dependent on HIV viral load [39]. In our study, the majority of HIV^+^ had undetectable viral loads, which may explain why PD-1 expression was not increased in these patients. It is unclear whether KLRG1 expression is also dependent on the viral load, and this could not be investigated in our study due to the low number of patients with detectable viral loads. These observations suggest that CD8^**+**^ T cells from treated HIV-infected patients appear to be functional despite the skewed differentiation and maturation. However, due to the limited number of viable cells and FACS lasers, we could not investigate the functionality directly by assessing functional markers like CD56; the co-expression of PD-1 and KLRG1, and co-expression with other inhibitory receptors like TIM-3. But we did find a positive association between KLRG1 and PD-1 expression. Investigating CD56 in the subsets could have yielded insight into the functionality of CD8^+^ T cells by assessing cytotoxicity [40]. Moreover, assessing TIM-3 expression as a marker of exhaustion could have yielded insight into the exhaustion of CD8^+^ T cells with cytotoxic effects (CD56^+^) as in Poonia et al. [40]. Co-expression of several inhibitory receptors may be necessary to affect cellular functions, and may be a prominent feature in chronic viral infections [41, 42]. However, the aim of this study was to assess the effect of immunosenescence and exhaustion in CD8^+^ T cells on age and age-related parameters, rather than CD8^+^ T cell functions. We therefore investigated KLRG1 and PD-1, as these have been shown to reflect CD8^+^ T cell senescence and exhaustion [8, 14].

KLRG1 expression in the subsets, but not in total CD8^**+**^ T cells, was influenced to a minor degree by age-related processes of metabolism, adipose tissue, and muscle. VAT and metabolic syndrome were associated with higher KLRG1 expression in CD28^+^ and CD28^−^ cells. KLRG1 expression in T_N_ cells was associated with high muscle mass. However, due to the wide confidence intervals for the associations with metabolic syndrome and *l*LMI, and to the small estimate of the association with VAT, further investigations are required to determine whether these associations are true, or whether they are artefacts. PD-1 expression was not influenced by age-related parameters. Moreover, we did not find obvious associations between CD8^**+**^ T cell phenotypes and inflammation, suggesting that highly differentiated, mature, senescent, or exhausted CD8^**+**^ T cells may not be major contributors to systemic IL-6 and suPAR levels.

In participants from this study, IL-6 and suPAR were associated with FMI, VAT and low *l*LMI [17]. These observations suggest that inflammation reflects age-related processes of adipose tissue redistribution and low muscle mass. In contrast, we did not find definite associations of immunosenescence and T cell exhaustion with age-related parameters or inflammation. This indicates that CD8^**+**^ T cell immunosenescence and exhaustion may not play major roles in inflammaging and age-related processes in HIV-infected patients.

This study has some limitations. Its cross-sectional nature does not allow for causal interpretation of the associations. Since only ART-treated patients were included, we could not determine whether our findings were a result of HIV-infection, or ART, or a combination. Moreover, due to difficulties in including treatment naïve patients, we were not able to include elite controllers for comparison. By restricting the inclusion criteria to men we aimed to limit variation in the immune and inflammatory variables due to menopause and menstrual cycle-related hormonal fluctuations [43]. Therefore, our findings are only representative for men. The lack of data on CMV-seropositivity of the participants may also be a limitation to the interpretation of the results. CMV seroprevalence is higher in HIV-infection (>75 %) than in the general population (40–50 %) [44]. Thus, CMV-seropositivity could also contribute to the skewed distribution of CD8^**+**^ T cells we observed in HIV^+^. Another limitation to the study is the likelihood of type I error due to multiple testing. We limited the number of analyses by grouping the CD8^+^ T cell subsets into larger groups based on relevant biological and functional features. Moreover, we based the interpretation of our data on other findings, and did not make definite conclusions from our findings that did not reach high statistical significance. The study population was relatively small due to a number of non-viable samples. We therefore did not have sufficient power to assess ageing parameters in the healthy Controls.

## Conclusions

We showed that despite effective treatment, HIV-infection is associated with a skewed distribution of CD8^+^ T cells towards more differentiated and mature phenotypes, suggestive of accelerated immune ageing. However, HIV-infection was not associated with higher KLRG1 or PD-1 expression on CD8^+^ T cells. Instead, the pattern of KLRG1 and PD-1 expression followed subset distribution–and to a minor degree, age. We did not find obvious relationships between CD8^**+**^ T cell phenotypes and age-related processes of inflammation, metabolism, adipose tissue and muscle. These findings suggest that in contrast to inflammation, immunosenescence appears to be highly dependent on HIV-infection and is only to a smaller extent associated with age-related parameters in well-treated HIV-infected patients. Moreover, CD8^**+**^ T cell differentiation, maturation, senescence, or exhaustion did not appear to be major causes of inflammaging in HIV-infected patients.

## Additional files

Additional file 1: Figure S1. CD8^+^ T cell maturation and differentiation in HIV^+^ with and without lipodystrophy, and Controls. (a) Proportions of CD8^+^ T cells in each maturation subset: naïve (T_N_), central memory (T_CM_), effector memory (T_EM_), or effector memory re-expressing CD45RA (T_EMRA_) (b) Proportions of CD8^+^ T cells in each differentiation subset: early differentiated (T_ED_), intermediate differentiated (T_ID_), late differentiated (T_LD_), or CD27^−^CD28^+^. HIV^+^ with lipodystrophy (Lipo) (*N* = 14), HIV^+^ without lipodystrophy (Non-lipo) (*N* = 29), and Controls (*N* = 10). Medians are shown as horizontal bars. *P*-values were determined using one-way ANOVA. (PDF 64 kb) Additional file 2: Figure S2. CD8^+^ T cell exhaustion and senescence in HIV^+^ with and without lipodystrophy, and Controls. (a) Proportions of PD-1^+^ cells in CD8^+^ T cell subsets and total CD8^+^ T cells in HIV^+^ with and without lipodystrophy, and Controls. HIV^+^ with lipodystrophy (Lipo) (*N* = 14), HIV^+^ without lipodystrophy (Non-lipo) (*N* = 29), and Controls (*N* = 10). (b) Proportions of KLRG1^+^ cells in CD8^+^ T cell subsets and total CD8^+^ T cells in HIV^+^ with and without lipodystrophy, and Controls. HIV^+^ with lipodystrophy (Lipo) (*N* = 8), HIV^+^ without lipodystrophy (Non-lipo) (*N* = 22), and Controls (*N* = 9). Medians are shown as horizontal bars. *P*-values were determined using one-way ANOVA. (PDF 98 kb) Additional file 3: Table S1. Linear regression analyses for the effect of HIV-infection on CD8^+^ T cell maturation and differentiation. ^†^Parameter was transformed using log_2_(x). Estimates and confidence intervals are back transformed using (2^β^-1) × 100, and shown as percent change in the outcome per unit increase of the covariate. The bold values are statistically significant at **P* < 0.05, ***P* < 0.01, ****P* < 0.001. Abbreviations: HOMA-IR: homeostatic model assessment of insulin resistance, IL-6: interleukin-6, *l*LMI: leg lean mass index, suPAR: soluble urokinase plasminogen activator receptor, T_CM_: central memory T cell, T_ED_: early differentiated T cell, T_EM_: effector memory T cell, T_EMRA_: effector memory T cell re-expressing CD45RA, T_ID_: intermediate differentiated T cell, T_LD_: late differentiated T cell, T_N_: naïve T cell, VAT: visceral adipose tissue. (XLSX 14 kb) Additional file 4: Table S2. Unadjusted and adjusted associations between CD8^+^ T cell subset sizes and ageing parameters in HIV^+^. Adjusted analyses are in grey. Age was adjusted for HIV-duration, ART-duration, and current ART; IL-6, suPAR, HOMA-IR, metabolic syndrome, VAT, and *l*LMI were adjusted for age, HIV-duration, ART-duration, and current ART. ^†^Parameters were transformed using log_2_(x). Estimates and confidence intervals are back transformed using (2^β^-1) × 100, and shown as percent change in the outcome per unit increase of the covariate. The bold values are statistically significant at **P* < 0.05, ***P* < 0.01, ****P* < 0.001. Abbreviations: HOMA-IR: homeostatic model assessment of insulin resistance, IL-6: interleukin-6, *l*LMI: leg lean mass index, suPAR: soluble urokinase plasminogen activator receptor, T_CM_: central memory T cell, T_ED_: early differentiated T cell, T_EM_: effector memory T cell, T_EMRA_: effector memory T cell re-expressing CD45RA, T_ID_: intermediate differentiated T cell, T_LD_: late differentiated T cell, T_N_: naïve T cell, VAT: visceral adipose tissue. (XLSX 16 kb) Additional file 5: Table S3. Unadjusted and adjusted associations between KLRG1^+^CD8^+^ T cell subsets and ageing parameters in HIV^+^. Adjusted analyses are in grey. Age was adjusted for HIV-duration, ART-duration, and current ART; IL-6, suPAR, HOMA-IR, metabolic syndrome, VAT, and *l*LMI were adjusted for age, HIV-duration, ART-duration, and current ART. ^†^Parameters were transformed using log_2_(x). Estimates and confidence intervals are back transformed using (2^β^-1) × 100, and shown as percent change in the outcome per unit increase of the covariate. The bold values are statistically significant at **P* < 0.05, ***P* < 0.01, ****P* < 0.001. Abbreviations: HOMA-IR: homeostatic model assessment of insulin resistance, IL-6: interleukin-6, KLRG1: killer cell lectin-like receptor G1, *l*LMI: leg lean mass index, suPAR: soluble urokinase plasminogen activator receptor, T_CM_: central memory T cell, T_ED_: early differentiated T cell, T_EM_: effector memory T cell, T_EMRA_: effector memory T cell re-expressing CD45RA, T_ID_: intermediate differentiated T cell, T_LD_: late differentiated T cell, T_N_: naïve T cell, VAT: visceral adipose tissue. (XLSX 16 kb) Additional file 6: Table S4. Unadjusted and adjusted associations between PD-1^+^CD8^+^ T cell groups and ageing parameters in HIV^+^. Adjusted analyses are in grey. Age was adjusted for HIV-duration, ART-duration, and current ART; IL-6, suPAR, HOMA-IR, metabolic syndrome, VAT, and *l*LMI were adjusted for age, HIV-duration, ART-duration, and current ART. ^†^Parameters were transformed using log_2_(x). Estimates and confidence intervals are back transformed using (2^β^-1) × 100, and shown as percent change in the outcome per unit increase of the covariate. Abbreviations: HOMA-IR: homeostatic model assessment of insulin resistance, IL-6: interleukin-6, PD-1: programmed death-1, *l*LMI: leg lean mass index, suPAR: soluble urokinase plasminogen activator receptor, T_CM_: central memory T cell, T_ED_: early differentiated T cell, T_EM_: effector memory T cell, T_EMRA_: effector memory T cell re-expressing CD45RA, T_ID_: intermediate differentiated T cell, T_LD_: late differentiated T cell, T_N_: naïve T cell, VAT: visceral adipose tissue. (XLSX 15 kb) Additional file 7: Table S5. Unadjusted and adjusted associations between PD-1^+^CD8^+^ T cell subsets and ageing parameters in HIV^+^. Adjusted analyses are in grey. Age was adjusted for HIV-duration, ART-duration, and current ART; IL-6, suPAR, HOMA-IR, metabolic syndrome, VAT, and *l*LMI were adjusted for age, HIV-duration, ART-duration, and current ART. ^†^Parameters were transformed using log_2_(x). Estimates and confidence intervals are back transformed using (2^β^-1) × 100, and shown as percent change in the outcome per unit increase of the covariate. Abbreviations: HOMA-IR: homeostatic model assessment of insulin resistance, IL-6: interleukin-6, PD-1: programmed death-1, *l*LMI: leg lean mass index, suPAR: soluble urokinase plasminogen activator receptor, T_CM_: central memory T cell, T_ED_: early differentiated T cell, T_EM_: effector memory T cell, T_EMRA_: effector memory T cell re-expressing CD45RA, T_ID_: intermediate differentiated T cell, T_LD_: late differentiated T cell, T_N_: naïve T cell, VAT: visceral adipose tissue. (XLSX 17 kb)

## Abbreviations

ART: antiretroviral therapy
BMI: body mass index
CMV: cytomegalovirus
FMI: fat mass index
HIV^+^: HIV-infected patient
HOMA-IR: homeostatic model assessment of insulin resistance
IL-6: interleukin-6
KLRG1: killer cell lectin-like receptor G1
*l*LMI: leg lean mass index
PD-1: programmed death-1
suPAR: soluble urokinase plasminogen activator receptor
T_CM_: central memory
T_ED_: early differentiated
T_EM_: effector memory
T_EMRA_: effector memory re-expressing CD45RA
T_ID_: intermediate differentiated
T_LD_: late differentiated
T_N_: naïve
VAT: visceral adipose tissue

## References

[1] Department of Economic and Social Affairs (Population Division), United Nations. Volume I Comprehensive Tables. In: World Population Prospects : The 2012 Revision. 2012. http://esa.un.org/unpd/wpp/publications/Files/WPP2012_Volume-I_Comprehensive-Tables.pdf. Accessed 25 Nov 2015.

[2] Singh T, Newman AB (2011). Inflammatory markers in population studies of aging. Ageing Res Rev.

[3] Franceschi C, Campisi J (2014). Chronic inflammation (inflammaging) and its potential contribution to age-associated diseases. J Gerontol A Biol Sci Med Sci.

[4] Deeks SG (2011). HIV Infection, Inflammation, Immunosenescence, and Aging. Annu Rev Med.

[5] Arnold CR, Wolf J, Brunner S, Herndler-Brandstetter D, Grubeck-Loebenstein B (2011). Gain and Loss of T Cell Subsets in Old Age—Age-Related Reshaping of the T Cell Repertoire. J Clin Immunol.

[6] Wikby A, Nilsson B-O, Forsey R, Thompson J, Strindhall J, Löfgren S (2006). The immune risk phenotype is associated with IL-6 in the terminal decline stage: Findings from the Swedish NONA immune longitudinal study of very late life functioning. Mech Ageing Dev.

[7] Akbar AN, Henson SM (2011). Are senescence and exhaustion intertwined or unrelated processes that compromise immunity?. Nat Rev Immunol.

[8] Henson SM, Franzese O, Macaulay R, Libri V, Azevedo RI, Kiani-Alikhan S (2009). KLRG1 signaling induces defective Akt (ser473) phosphorylation and proliferative dysfunction of highly differentiated CD8+ T cells. Blood.

[9] Effros RB, Dagarag M, Spaulding C, Man J (2005). The role of CD8+ T-cell replicative senescence in human aging. Immunol Rev.

[10] Pathai S, Bajillan H, Landay AL, High KP (2014). Is HIV a Model of Accelerated or Accentuated Aging?. J Gerontol A Biol Sci Med Sci.

[11] Guaraldi G, Orlando G, Zona S, Menozzi M, Carli F, Garlassi E (2011). Premature Age-Related Comorbidities Among HIV-Infected Persons Compared With the General Population. Clin Infect Dis.

[12] Emu B, Moretto WJ, Hoh R, Krone M, Martin JN, Nixon DF (2014). Composition and Function of T Cell Subpopulations Are Slow to Change Despite Effective Antiretroviral Treatment of HIV Disease. PLoS ONE.

[13] Neuhaus J, Jacobs DR, Baker JV, Calmy A, Duprez D, La Rosa A (2010). Markers of Inflammation, Coagulation, and Renal Function Are Elevated in Adults with HIV Infection. J Infect Dis.

[14] Trautmann L, Janbazian L, Chomont N, Said EA, Gimmig S, Bessette B (2006). Upregulation of PD-1 expression on HIV-specific CD8+ T cells leads to reversible immune dysfunction. Nat Med.

[15] Andersen O, Haugaard SB, Andersen UB, Friis-Møller N, Storgaard H, Vølund A (2003). Lipodystrophy in human immunodeficiency virus patients impairs insulin action and induces defects in β-cell function. Metabolism.

[16] Bernasconi E, Boubaker K, Junghans C, Flepp M, Furrer HJ, Haensel A (2002). Swiss HIV Cohort Study. Abnormalities of body fat distribution in HIV-infected persons treated with antiretroviral drugs: The Swiss HIV Cohort Study. J Acquir Immune Defic Syndr.

[17] Langkilde A, Petersen J, Henriksen JH, Jensen FK, Gerstoft J, Eugen-Olsen J (2015). Leptin, IL-6, and suPAR reflect distinct inflammatory changes associated with adiposity, lipodystrophy and low muscle mass in HIV-infected patients and Controls. Immun Ageing.

[18] Langkilde A, Andersen O, Henriksen JH, Langberg H, Petersen J, Eugen-Olsen J (2015). Assessment of in situ adipose tissue inflammation by microdialysis. Clin Physiol Funct Imaging.

[19] Kotecha N, Krutzik PO, Irish JM. Web-Based Analysis and Publication of Flow Cytometry Experiments. Curr Protoc Cytom. 2010;Chapter 10:Unit10.17. 10.1002/0471142956.cy1017s53 PMC4208272 20578106

[20] Henson SM, Akbar AN (2009). KLRG1–more than a marker for T cell senescence. Age.

[21] Appay V, Dunbar PR, Callan M, Klenerman P, Gillespie GM, Papagno L (2002). Memory CD8+ T cells vary in differentiation phenotype in different persistent virus infections. Nat Med.

[22] Solana R, Tarazona R, Aiello AE, Akbar AN, Appay V, Beswick M (2012). CMV and Immunosenescence: from basics to clinics. Immun Ageing.

[23] Heymsfield SB, Wang Z, Baumgartner RN, Ross R (1997). Human Body Composition: Advances in Models and Methods. Annu Rev Nutr.

[24] Matthews DR, Hosker JP, Rudenski AS, Naylor BA, Treacher DF, Turner RC (1985). Homeostasis model assessment: insulin resistance and beta-cell function from fasting plasma glucose and insulin concentrations in man. Diabetologia.

[25] Alberti KGMM, Eckel RH, Grundy SM, Zimmet PZ, Cleeman JI, Donato KA (2009). Harmonizing the Metabolic Syndrome A Joint Interim Statement of the International Diabetes Federation Task Force on Epidemiology and Prevention; National Heart, Lung, and Blood Institute; American Heart Association; World Heart Federation; International Atherosclerosis Society; and International Association for the Study of Obesity. Circulation.

[26] Rosignoli G, Lim CH, Bower M, Gotch F, Imami N (2009). Programmed death (PD)-1 molecule and its ligand PD-L1 distribution among memory CD4 and CD8 T cell subsets in human immunodeficiency virus-1-infected individuals. Clin Exp Immunol.

[27] Derhovanessian E, Maier AB, Hähnel K, Beck R, de Craen AJM, Slagboom EP (2011). Infection with cytomegalovirus but not herpes simplex virus induces the accumulation of late-differentiated CD4+ and CD8+ T-cells in humans. J Gen Virol.

[28] Koch S, Larbi A, Derhovanessian E, Özcelik D, Naumova E, Pawelec G (2008). Multiparameter flow cytometric analysis of CD4 and CD8 T cell subsets in young and old people. Immun Ageing.

[29] Ye P, Kirschner DE, Kourtis AP (2004). The thymus during HIV disease: role in pathogenesis and in immune recovery. Curr HIV Res.

[30] Herasimtschuk AA, Hansen BR, Langkilde A, Moyle GJ, Andersen O, Imami N (2013). Low-dose growth hormone for 40 weeks induces HIV-1-specific T cell responses in patients on effective combination anti-retroviral therapy. Clin Exp Immunol.

[31] Finzi D, Hermankova M, Pierson T, Carruth LM, Buck C, Chaisson RE (1997). Identification of a Reservoir for HIV-1 in Patients on Highly Active Antiretroviral Therapy. Science.

[32] Appay V, Sauce D (2008). Immune activation and inflammation in HIV-1 infection: causes and consequences. J Pathol.

[33] Semba RD, Margolick JB, Leng S, Walston J, Ricks MO, Fried LP (2005). T cell subsets and mortality in older community-dwelling women. Exp Gerontol.

[34] Spielmann G, Johnston CA, O’Connor DP, Foreyt JP, Simpson RJ (2014). Excess body mass is associated with T cell differentiation indicative of immune ageing in children. Clin Exp Immunol.

[35] Erlandson KM, Allshouse AA, Jankowski CM, Lee EJ, Rufner KM, Palmer BE (2013). Association of Functional Impairment with Inflammation and Immune Activation in HIV Type 1–Infected Adults Receiving Effective Antiretroviral Therapy. J Infect Dis.

[36] Wallet MA, Buford TW, Joseph AM, Sankuratri M, Leeuwenburgh C, Pahor M (2015). Increased inflammation but similar physical composition and function in older-aged, HIV-1 infected subjects. BMC Immunol.

[37] Legat A, Speiser DE, Pircher H, Zehn D, Fuertes Marraco SA (2013). Inhibitory Receptor Expression Depends More Dominantly on Differentiation and Activation than «Exhaustion» of Human CD8 T Cells. Front Immunol.

[38] Sauce D, Almeida JR, Larsen M, Haro L, Autran B, Freeman GJ (2007). PD-1 expression on human CD8 T cells depends on both state of differentiation and activation status. AIDS.

[39] Cockerham LR, Jain V, Sinclair E, Glidden DV, Hartogenesis W, Hatano H (2014). Programmed death-1 expression on CD4+ and CD8+ T cells in treated and untreated HIV disease. AIDS.

[40] Poonia B, Pauza CD (2014). Levels of CD56 + TIM-3- Effector CD8 T Cells Distinguish HIV Natural Virus Suppressors from Patients Receiving Antiretroviral Therapy. PLoS ONE.

[41] Peretz Y, He Z, Shi Y, Yassine-Diab B, Goulet JP, Bordi R (2012). CD160 and PD-1 Co-Expression on HIV-Specific CD8 T Cells Defines a Subset with Advanced Dysfunction. PLoS Pathog.

[42] Bengsch B, Seigel B, Ruhl M, Timm J, Kuntz M, Blum HE (2010). Coexpression of PD-1, 2B4, CD160 and KLRG1 on Exhausted HCV-Specific CD8+ T Cells Is Linked to Antigen Recognition and T Cell Differentiation. PLoS Pathog.

[43] Lee S, Kim J, Jang B, Hur S, Jung U, Kil K (2010). Fluctuation of peripheral blood T, B, and NK cells during a menstrual cycle of normal healthy women. J Immunol.

[44] Lang DJ, Kovacs AA, Zaia JA, Doelkin G, Nilanhad JC, Aledort L (1989). Seroepidemiologic studies of cytomegalovirus and Epstein-Barr virus infections in relation to human immunodeficiency virus type 1 infection in selected recipient populations. Transfusion Safety Study Group. J Acquir Immune Defic Syndr.

