# Supplementary figures and images for: Immunosenescence of the CD8+ T cell compartment is associated with HIV-infection, but only weakly reflects age-related processes of adipose tissue, metabolism, and muscle in antiretroviral therapy-treated HIV-infected patients and controls
Source: BMC Immunol. 2015 Nov 26;16:72. doi: 10.1186/s12865-015-0136-6 (PMC4661963; doi:10.1186/s12865-015-0136-6)

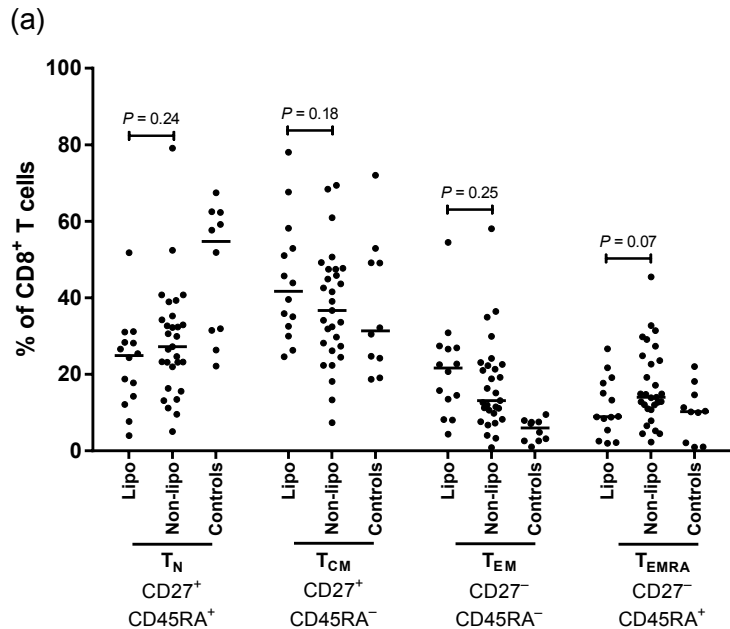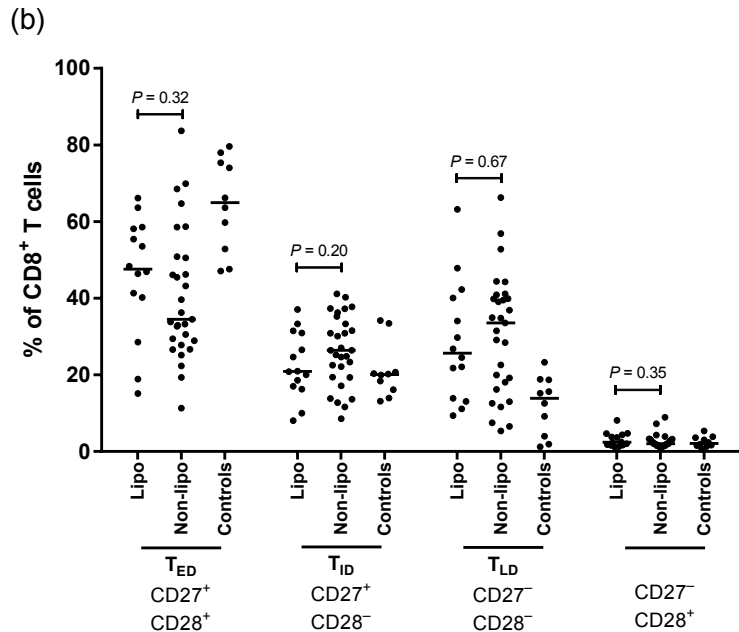

Supplement: Additional file 1: Figure S1. — CD8+ T cell maturation and differentiation in HIV+ with and without lipodystrophy, and Controls. (a) Proportions of CD8+ T cells in each maturation subset: naïve (TN), central memory (TCM), effector memory (TEM), or effector memory re-expressing CD45RA (TEMRA) (b) Proportions of CD8+ T cells in each differentiation subset: early differentiated (TED), intermediate differentiated (TID), late differentiated (TLD), or CD27−CD28+. HIV+ with lipodystrophy (Lipo) (N = 14), HIV+ without lipodystrophy (Non-lipo) (N = 29), and Controls (N = 10). Medians are shown as horizontal bars. P-values were determined using one-way ANOVA. (PDF 64 kb) [file 12865_2015_136_MOESM1_ESM.pdf]

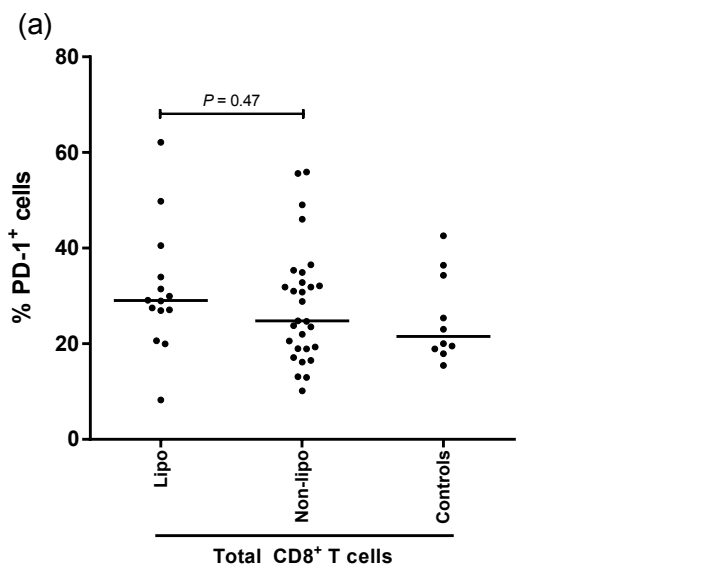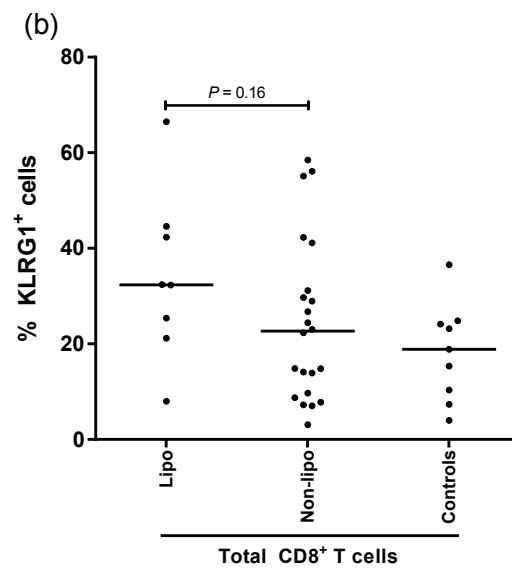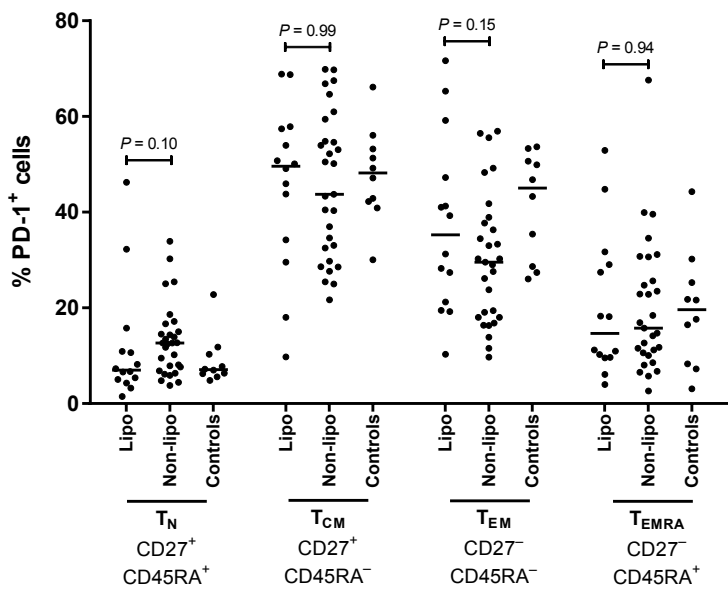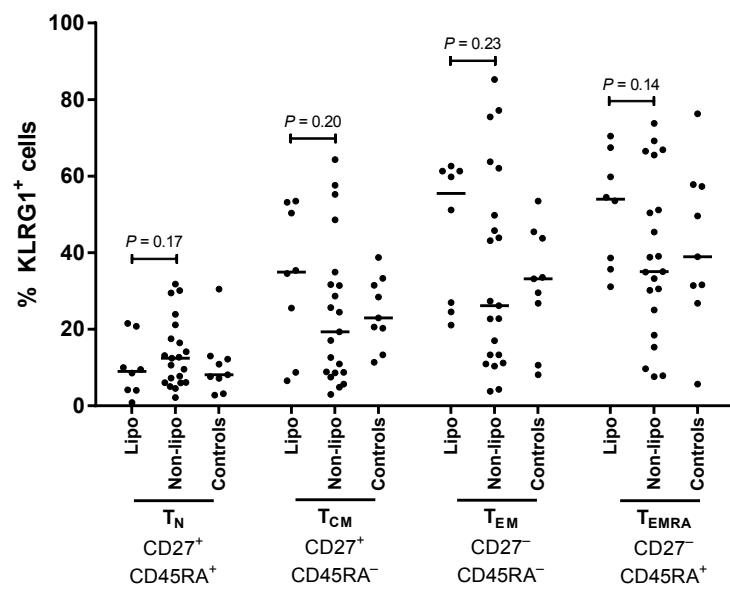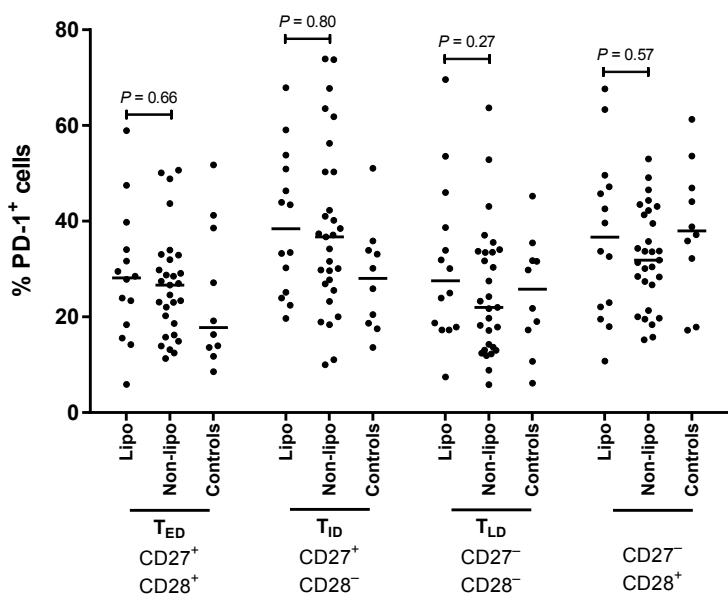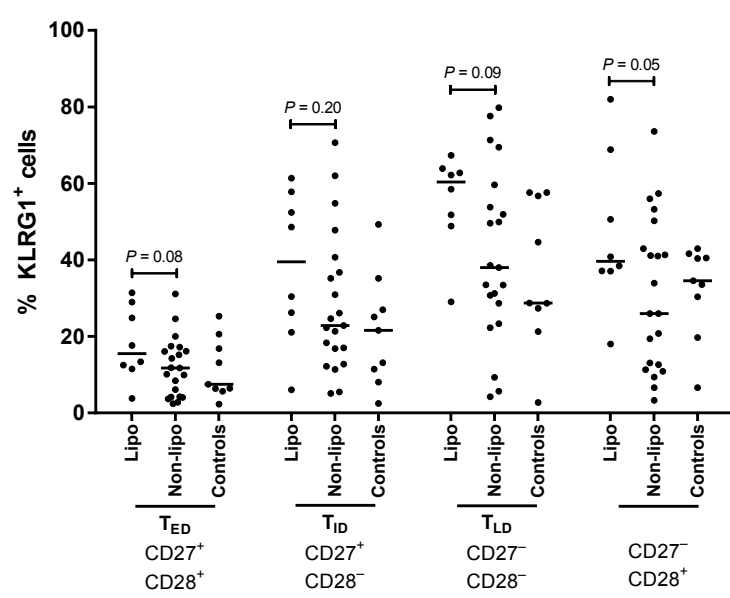

Supplement: Additional file 2: Figure S2. — CD8+ T cell exhaustion and senescence in HIV+ with and without lipodystrophy, and Controls. (a) Proportions of PD-1+ cells in CD8+ T cell subsets and total CD8+ T cells in HIV+ with and without lipodystrophy, and Controls. HIV+ with lipodystrophy (Lipo) (N = 14), HIV+ without lipodystrophy (Non-lipo) (N = 29), and Controls (N = 10). (b) Proportions of KLRG1+ cells in CD8+ T cell subsets and total CD8+ T cells in HIV+ with and without lipodystrophy, and Controls. HIV+ with lipodystrophy (Lipo) (N = 8), HIV+ without lipodystrophy (Non-lipo) (N = 22), and Controls (N = 9). Medians are shown as horizontal bars. P-values were determined using one-way ANOVA. (PDF 98 kb) [file 12865_2015_136_MOESM2_ESM.pdf]
